# Effect of the Microstructure and Distribution of the Second Phase on the Stress Corrosion Cracking of Biomedical Mg-Zn-Zr-xSr Alloys

**DOI:** 10.3390/ma11040551

**Published:** 2018-04-03

**Authors:** Lianxi Chen, Yinying Sheng, Xiaojian Wang, Xueyang Zhao, Hui Liu, Wei Li

**Affiliations:** 1 Institute of Advanced Wear & Corrosion Resistant and Functional Materials, Jinan University, Guangzhou 510632, China; chenlianxigood@163.com (L.C.); iwrmsyy@163.com (Y.S.); Hc_sunboy@163.com (X.Z.); liuhuijnu@163.com (H.L.); 2 National Joint Engineering Center of High-Performance Wear-Resistant Metallic Materials, Guangzhou 510632, China

**Keywords:** biodegradable, magnesium alloys, microstructure, second phase, stress corrosion cracking

## Abstract

The stress corrosion cracking (SCC) properties of the bi-directional forged (BDF) Mg-4Zn-0.6Zr-xSr (ZK40-xSr, x = 0, 0.4, 0.8, 1.2, 1.6 wt %) alloys were studied by the slow strain rate tensile (SSRT) testing in modified simulated body fluid (m-SBF). The average grain size of the BDF alloys were approximately two orders of magnitude smaller than those of the as-cast alloys. However, grain refinement increased the hydrogen embrittlement effect, leading to a higher SCC susceptibility in the BDF ZK40-0/0.4Sr alloys. Apart from the grain refinements effect, the forging process also changed the distribution of second phase from the net-like shape along the grain boundary to a uniformly isolated island shape in the BDF alloys. The SCC susceptibility of the BDF ZK40-1.2/1.6Sr alloys were lower than those of the as-cast alloys. The change of distribution of the second phase suppressed the adverse effect of Sr on the SCC susceptibility in high Sr–containing magnesium alloys. The results indicated the stress corrosion behavior of magnesium alloys was related to the average grain size of matrix and the distribution and shape of the second phase.

## 1. Introduction

As biodegradable implant materials, magnesium (Mg) and its alloys have recently attracted increasing research attentions for their ability to completely degrade in the body after fulfilling their temporary function [1,2]. Mg-based alloys have a close elasticity modulus to cortical bone, which could decrease or avoid the “stress shielding effect”. The degradation products of Mg are essential to human metabolism system as a cofactor for many enzymes and are reported to benefit the growth and healing of many tissues [3]. Moreover, excessive Mg ions can be excreted outside the body through the excretion system [4,5]. In comparison with biodegradable polymers, Mg alloys exhibit good mechanical strength. The tensile strength of human bone, PLGA, and Mg alloys are 110–130, 13.9–16.7, and 150–290 MPa, respectively [6]. These make Mg and Mg alloys suitable candidates for orthopedic implants [7] (e.g., bone plate and screw) and cardiovascular [8] (e.g., stent) applications.

Despite the above-mentioned advantages, Mg alloys tend to degrade rapidly in the physiological environment, thereby losing their mechanical properties before completing their service life. This limits their applications as implant devices [9,10,11]. Many efforts have been carried out to control the degradation process of Mg alloys, by designing novel corrosion resistant Mg alloys. For instance, Mg-Zn-Ca [3], Mg-Zn-Zr-Re [12], Mg-Nd-Zn-Zr [13], and Mg-Sr [14] alloys have been designed. The degradation rates of these new alloys were reported to be significantly reduced as compared to pure Mg. Animal tests showed the new bone formation around these new magnesium alloys [15]. Another approach to control the degradation process of Mg alloys is through surface modifications [16,17].

For orthopedic and cardiovascular implants, materials are often also subjected to complex stress environments in vivo, including tension, compression, bending, and cyclic mechanical loadings [18,19,20]. For example, a bone plate needs to bear certain tensile stress for connecting the fractured bones together [21]. A cardiovascular stent is subjected to cyclic loading due to heart beat or the residual stress at the deformed locations after expansion [18]. The presence of complex stresses along with a chloride-containing environment may lead to unexpected fracture, as a result of the effect of stress assisted degradation cracking (stress corrosion cracking, SCC) [22,23,24] or corrosion fatigue (CF) [25]. Stress corrosion failure was reported in traditional non-degradable metallic implants such as titanium alloys and stainless steel [26,27]. Mg alloys were reported to be highly sensitive to SCC in the Cl-containing environment (even in deionized water) [22]. If the SCC fracture happens, there are some serious consequences, including the troublesome removal of failed implant materials and painful irritation or inflammation of the surrounding tissues [28]. 

Choudhary and Raman [29] studied the SCC properties of AZ91D Mg alloy in air and in modified simulated body fluid (m-SBF). Their results suggested a mixed mechanism of hydrogen embrittlement and anodic dissolution for the SCC susceptibility in AZ91D, confirmed by the presence of trans-granular SCC (TGSCC) over the fractured features. Winzer et al. [30] investigated the SCC susceptibility of Mg-Al alloys. Their results concluded that AZ31, AM30 and AZ91 were sensitive to the SCC in distilled water, and they attributed it to the diffusivity of hydrogen in the α-matrix. The aluminum-free Mg alloys, such as ZX50, WZ21 and WE43, were reported susceptible to SCC in m-SBF and confirmed by fracture morphology features of TGSCC and/or intergranular SCC (IGSCC) [31]. The Mg alloys containing biocompatible elements were also such as reported suffering from SCC in m-SBF, such as Zn- and Ca-containing Mg alloys MgZn1Ca0.3 (ZX10) [32], and MgZn3Ca1 (ZX31) [33]. 

In our previous research, we studied the effect of Sr addition on the microstructure, degradation rate and SCC susceptibility of a newly designed biomedical Mg-4Zn-0.6Zr-xSr alloys (ZK40-xSr, x = 0, 0.4, 0.8, 1.2, 1.6) [34]. Sr addition was found improving the mechanical properties and corrosion resistance in thealloys [14]. The addition of Sr was also shown promoted the growth of osteoblasts and prevented cardiovascular disease [35,36,37,38,39]. It was shown that the addition of Sr reduced the average grain size and increased the degradation rate and the SCC susceptibility of the as-cast ZK40-xSr alloys. Many studies focused on the SCC of the coarse grain or refined magnesium alloys, such as AZ91 [29], AZ31 [40], and Mg-X alloys [41]. Both the grain size and the distribution of second phase had effects on the SCC susceptibility of Mg alloys. However, the mechanism behind this needs further investigation. The purpose of this work is to investigate the effect of grain refinement and homogenized second phase distribution on the SCC of the forged ZK40-xSr alloys.

## 2. Materials and Methods

### 2.1. Materials and Specimens

The as-cast Mg-Zn-Zr-xSr alloys (Mg-4 wt % Zn-0.6 wt % Zr-xSr, ZK40-xSr, x = 0, 0.4, 0.8, 1.2, 1.6 wt %) were produced according to previously procedure [34]. The actual chemical compositions of the as-cast ZK40-xSr alloys were presented in our previously publication [34]. 

The forged ZK40-xSr alloys were produced by bi-directional forging (BDF) process. The as-cast ingots were preheated at 300 °C for 3 h and homogenized at 375 °C for 16 h, then cooled down to 60 °C in water. Rectangular samples (sample size: x × y × z = 21 × 24 × 60 mm^3^) were machined from the ingots. The forging direction was vertical to the longest side of the rectangular samples. The forging axis during BDF process was changed by 90°, i.e., x → y → x → y → x. The forging processes were carried out on four-column hydraulic press (FCHP; YD32-315T, Tengzhou Hydraulic Machinery Factory, Tengzhou, Shandong, China) with an initial forging speed of 10 mm·s^−1^ after preheating in a muffle furnace at 375 °C for 15 min. A pass strain of ∆*ε* = 0.2, and 10 forging passes were employed. The BDF specimens was carried out to an accumulated strain of ∆*ε* = 2. The rectangular sample was drawn in the z direction. The BDF samples were reheated at 375 °C for 10 min between each of the forging passes. After the forging process, the BDF samples were reheated at 375 °C for 5 min to eliminate residual stress, followed by air cooling. 

The microstructure was observed in the center of the BDF samples using optical microscopy (OM, DMILM, Leica, Wetzlar, Germany). The average grain size was measured using the linear intercept method [34]. Specimens were etched with acetic picral (5 g picric acid, 10 mL acetic acid, 70 mL ethanol and 20 mL distilled water) for OM observation. The phases in the BDF ZK40-xSr alloys were identified using an X-ray diffractometer (XRD, D/Max-2400, Rigaku, Tokyo, Janpan, (20 mA, 36 kV, Cu-Kα radiation; scanning angle 2θ = 10–90°, rate = 4°·min^−1^)).

### 2.2. Degradation Behavior

Degradation tests were conducted in modified simulated body fluid (m-SBF) at 37 ± 1 °C. The chemical compositions of the m-SBF was 0.504 g NaHCO_3_, 0.230 g K_2_HPO_4_·3H_2_O, 5.403 g NaCl, 0.426 g KCl, 0.311 g MgCl_2_·6H_2_O, 17.892 g HEPES (2-(4-(2-hydroxyethyl)-1-piperazinyl) ethanesulfonic acid), 0.072 g Na_2_SO_4_, 0.293 g CaCl_2_ and 15 mL of a 1 mol·L^−1^ NaOH solution [42]. Three duplicate samples were performed in m-SBF to evaluate the degradation behavior of the alloys. The rectangular (5 × 10 × 10 mm) specimens, machined from the center of the BDF alloys, mounted in epoxy resin with a work exposed area of 1 cm^2^, were ground using 400, 600, 800, 1200, 1500, and 2000 # SiC papers and ultrasonically washed in deionized water and acetone. The ratio of the specimen work surface area (cm^2^) to the volume of m-SBF (mL) was 1:80. M-SBF was refreshed every 48 h in order to mimic the metabolism environments. The volume of H_2_ evolution was measured using an apparatus as described in the literature [43]. 

Electrochemical tests were conducted in m-SBF at 37 ± 1 °C via an electrochemical workstation (PARSTAT 4000, AMETEK, San Diego, CA, USA). The potentiodynamic polarization (Tafel) was conducted using a three-electrode cell, with the specimen as the working electrode, a platinum sheet as the counter electrode and a saturated calomel electrode (SCE) as the reference electrode. The potentiodynamic polarization tests were measured at a scanning rate of 0.5 mV·s^−1^ (from −0.25 to 0.25 V, referred to the open circuit point) and analyzed by software VersaStudio (AMETEK, San Diego, California, USA).

### 2.3. Slow Strain Rate Tensile Test

Stress corrosion cracking (SCC) susceptibility of the as-cast and BDF Mg alloys was measured using slow strain rate tensile (SSRT) tests in air and in m-SBF. Round tensile specimens with a 16 mm gauge length and a 3 mm diameter were machined from the center of the BDF samples so that the tensile direction became parallel to the z axis. The tensile specimens were grounded up to 2500 # SiC papers. The strain rate for SSRT testes measured in air or in m-SBF was 10^−6^·s^−1^ on a universal testing machine (UTM, CMT5504, SANS, Shenzhen, Guangdong, China) [44]. Three duplicate samples were performed to measure the SCC susceptibility of the as-cast and BDF alloys in air and m-SBF, respectively. The SCC susceptibility indices (*I_SCC_*) of the as-cast and BDF alloys were evaluated using the ultimate tensile strength (UTS) and elongation to failure (*ε*). The *I_SCC_* is defined as [45]

(1) $$I_{\mathrm{SCC}}=1-\frac{{\mathrm{UTS}}_{\text{in m-SBF}}(1+\varepsilon_{\text{in m-SBF}})}{{\mathrm{UTS}}_{\mathrm{in}\;\mathrm{air}}(1+\varepsilon_{\mathrm{in}\;\mathrm{air}})},$$

Lower value of *I_SCC_* represents lesser SCC susceptibility. If *I_SCC_* close to zero, it indicates the Mgalloys are immune to SCC. The fracture features of the BDF alloys were washed in chromic acid, cleaned with deionized water, and observed under scanning electron microscope (SEM; Phenom XL, PANalytical B.V., Almelo, Netherlandsy) equipped with energy-dispersive spectrometer (EDS) after the SSRT tests in air or m-SBF. 

## 3. Results

### 3.1. Microstructure and Compositions

**Figure 1:**
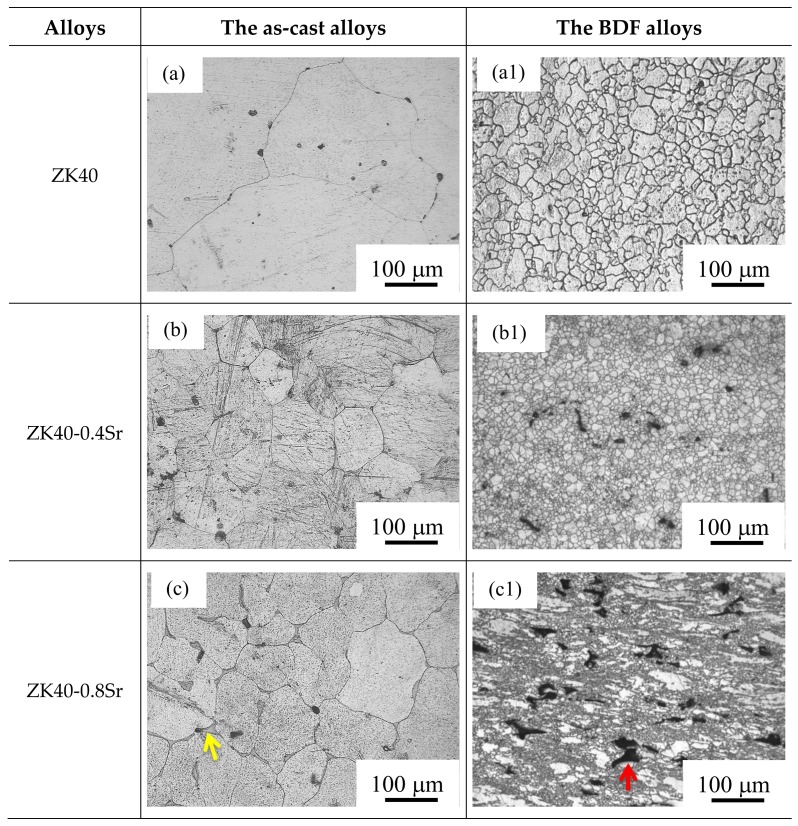

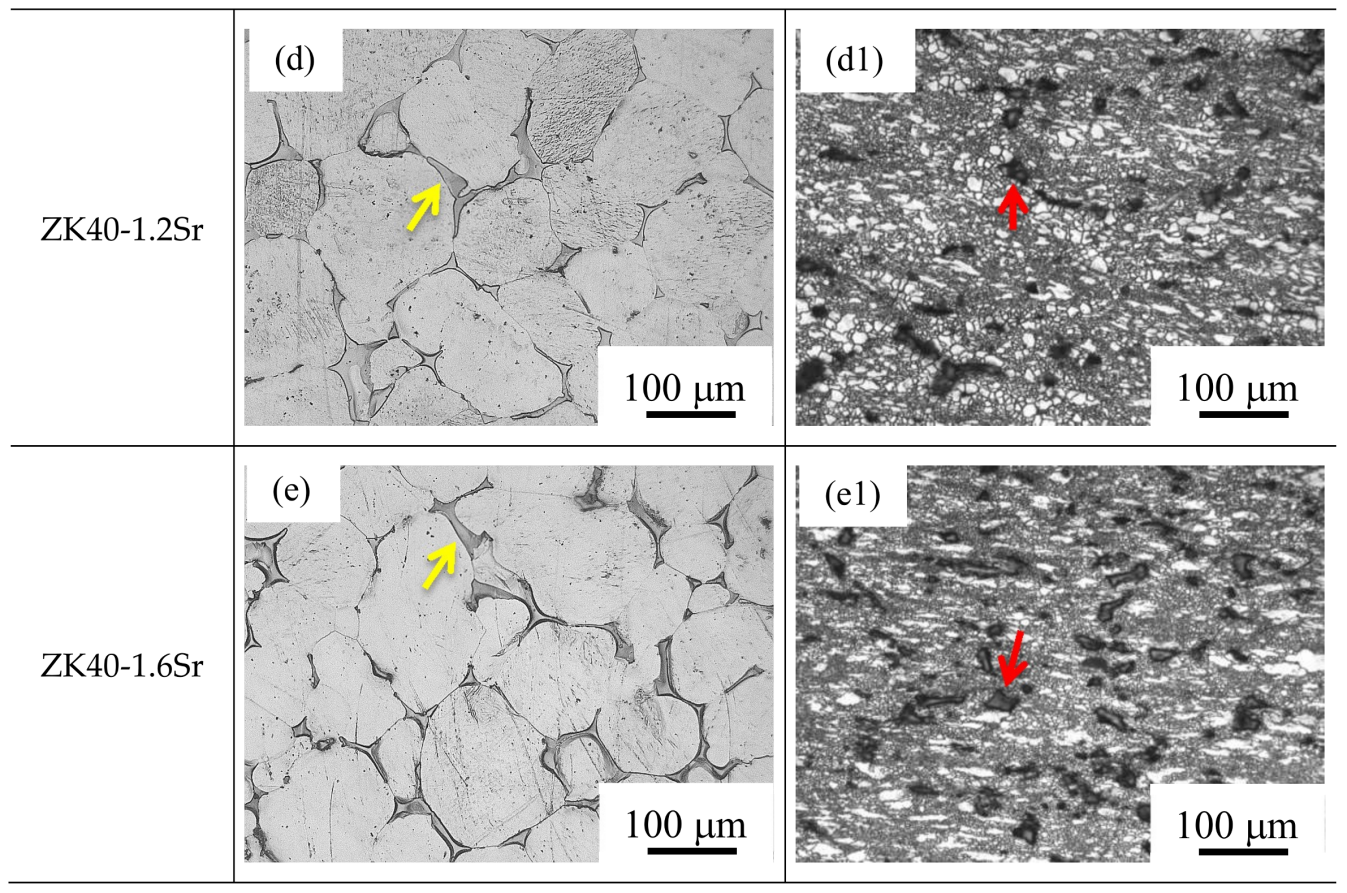
Optical microscopy images of the as-cast and bi-directional forging (BDF) ZK40-xSr alloys: (**a**,**a1**) ZK40; (**b**,**b1**) ZK40-0.4Sr; (**c**,**c1**) ZK40-0.8Sr; (**d**,**d1**) ZK40-1.2Sr; (**e**,**e1**) ZK40-1.6Sr.

Figure 1 shows the microstructure of the as-cast and bi-directional forging (BDF) ZK40-xSr alloys. The microstructure of the forged samples was different to that of the as-cast alloys. The grains of the BDF ZK40-xSr alloys became much finer and was distributed more homogeneously (as shown in Figure 1a,a1,b1). The shape of the second phase changed from lath line-like or net-like shape (as shown in Figure 1c,d,e by the yellow arrow) to island-like or ellipsoidal shape (Figure 1c1,d1,e1 by the red arrow) and not spread along the grain boundaries (as shown in Figure 1b–e). The size of the second phase was slightly bigger than the grain size of the matrix. The forging process refined the grains and changed the shape and distribution of the second phase.

**Figure 2:**
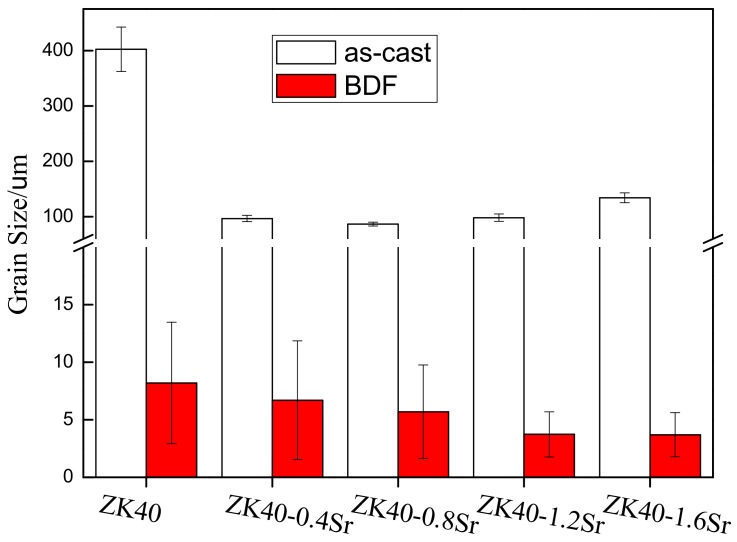
The comparison of the average grain size of the as-cast and BDF ZK40-xSr alloys.

The average grain size of the as-cast and BDF ZK40-xSr alloys was calculated using the linear intercept method (as shown in Figure 2). The average grain size of the as-cast ZK40-xSr (x = 0, 0.4, 0.8, 1.2, 1.6 wt %) alloys were 402.3 ± 40.2, 96.7 ± 5.6, 86.6 ± 3.4, 98.3 ± 6.7, and 134.1 ± 8.9 µm, respectively. The average grain size of the BDF ZK40-xSr alloys were 8.2 ± 5.3, 6.7 ± 5.2, 5.7 ± 4.1, 3.73 ± 2.0, and 3.7 ± 1.9 µm, respectively. The average grain size of the BDF alloys decreased with the increasing of Sr content and was less than 10 µm, reduced by almost two orders of magnitude compared to that of the as-cast alloys. The effect of Sr addition on limiting the growth of recrystallized grains in the plastic deformation was also demonstrated.

**Figure 3:**
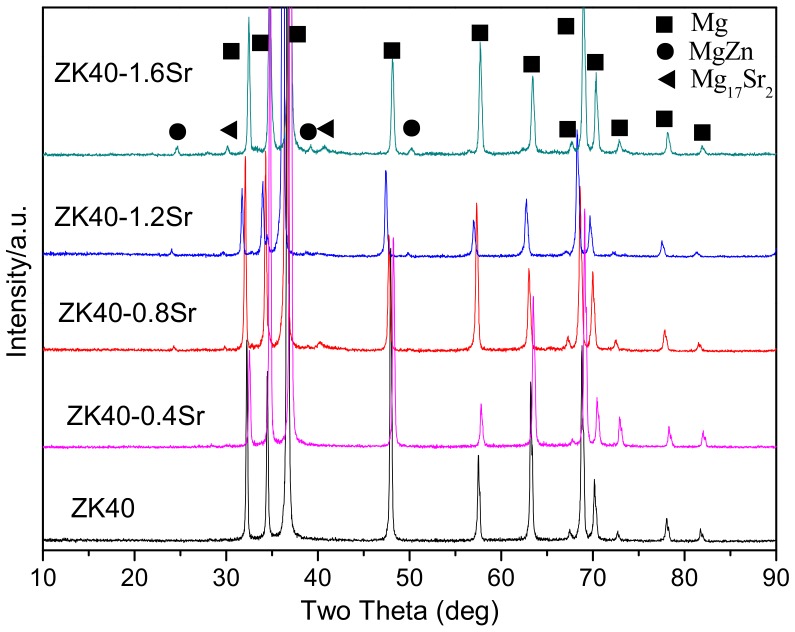
X-ray diffractometer (XRD) patterns of the BDF ZK40-xSr alloys.

Figure 3 shows the constituent phases of the BDF alloys. The phases in the BDF alloys were similar to the as-cast alloys α-Mg (ICDD JCPDS No. 35-0821), MgZn (ICDD JCPDS No. 40-1334), and Mg_17_Sr_2_ (ICDD JCPDS No. 18-1275). The Mg_17_Sr_2_ phase could not be detected in the ZK40-xSr (x ≤ 0.8 wt % Sr) with the low content of Sr. The peak intensities of Mg_17_Sr_2_ increased with the increase of Sr addition (x > 0.8 wt % Sr).

### 3.2. Biodegradation Behavior

**Figure 4:**
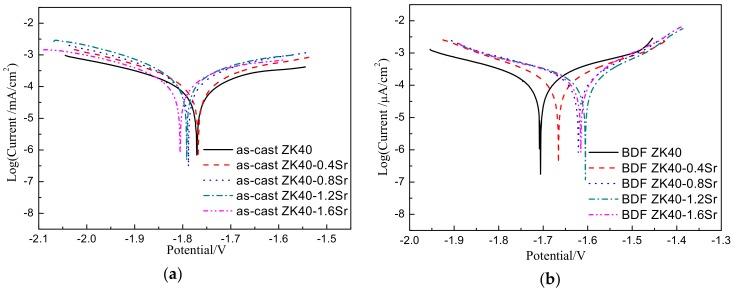
Potentiodynamic polarization curves of the as-cast (**a**) and BDF ZK40-xSr alloys (**b**) in modified simulated body fluid (m-SBF) at 37 °C.

Figure 4 shows the potentiodynamic polarization (Tafel) curves of the as-cast and BDF ZK40-xSr alloys. The corresponding values of corrosion current density and corrosion potential of the Tafel curves measured using VersaStudio software are listed in Table 1. Comparing to the as-cast alloys, the corrosion current density of the BDF alloys decreased, and the corrosion potential shifted positively. The lowest corrosion current density in as-cast alloys was ZK40 alloy (0.255 mA·cm^−2^), while that of the BDF alloys was ZK40-0.4Sr alloy (0.195 mA·cm^−2^). The results suggested Sr had less effect on increasing the degradation rate in the forged samples, as compared to the as-cast samples.

The values of degradation rate, calculated from the polarization curves and H_2_ evolution, are listed in Table 1. The lowest degradation rate measured by H_2_ volume in as-cast alloys was ZK40 alloy (4.59 ± 0.30 mm·y^−1^ by H_2_ evolution), while that of BDF alloys was ZK40-0.4Sr alloy (3.51 ± 0.19 mm·y^−1^ by H_2_ evolution). The degradation rate of the as-cast ZK40-xSr alloys increased significantly with the increase of Sr content, while that of the BDF alloys slightly fluctuated. The degradation rates calculated by the polarization curves and that of the H_2_ evolution experiments supported each other.

### 3.3. Slow Strain Rate Testing

**Figure 5:**
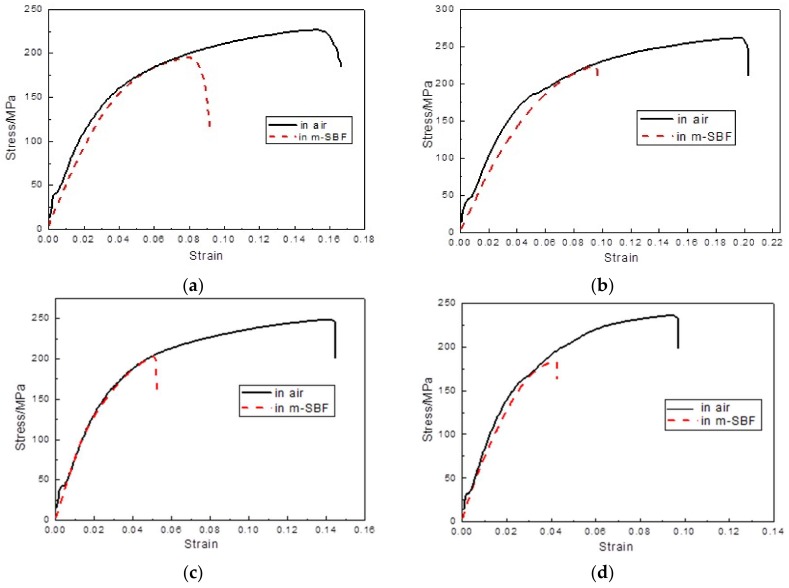

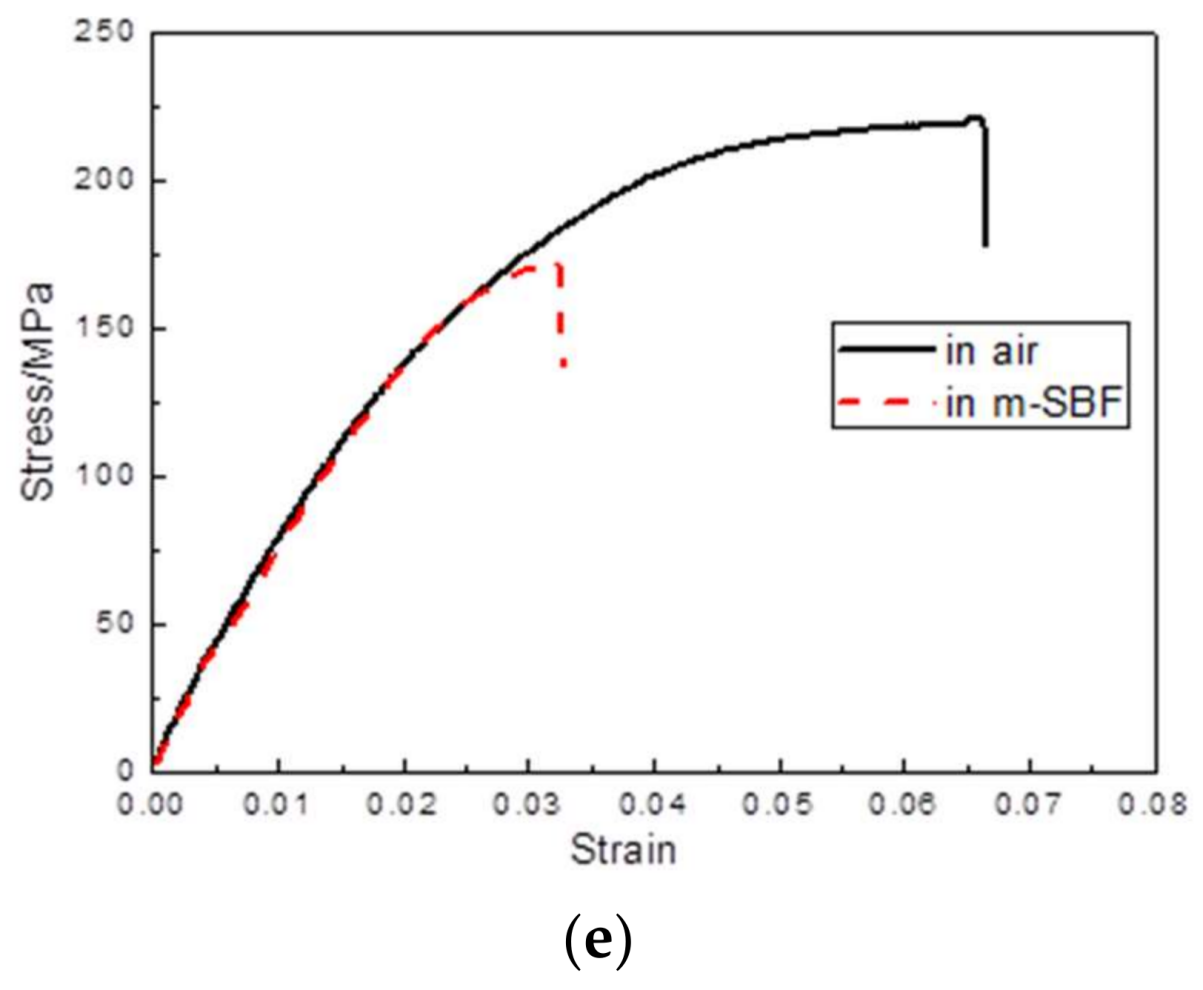
Tensile stress-strain curves of the BDF ZK40-xSr alloys tested in air and in m-SBF at 37 ± 1 °C. (**a**) ZK40; (**b**) ZK40-0.4Sr; (**c**) ZK40-0.8Sr; (**d**) ZK40-1.2Sr; (**e**) ZK40-1.6Sr.

Figure 5 shows the stress-strain curves of the BDF alloys conducted in air and in m-SBF. The results of the ultimate tensile strength (UTS), elongation to failure (*ε*) and stress corrosion cracking susceptibility indices (*I_SCC_*) calculated from the stress-strain curves are listed in Table 2. The ultimate tensile strength of the BDF alloys tested in air was all above 220 MPa. The elongation to failure of the BDF alloys decreased from 19.6 ± 0.7% to 6.6 ± 0.3%, with the increase of Sr content. The optimum mechanical properties were 261.1 ± 6.3 MPa and 19.6 ± 0.7% in air, 224.2 ± 7.7 MPa, and 9.5 ± 0.5% in m-SBF for the BDF ZK40-0.4Sr alloy.

**Figure 6:**
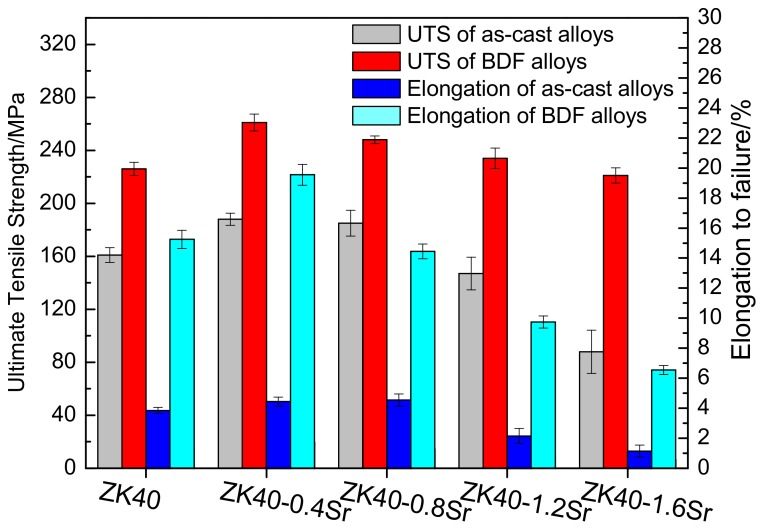
Comparison of the ultimate tensile strength (UTS) and elongation to failure (*ε*) of the as-cast and BDF ZK40-xSr alloys in air.

Figure 6 shows the elongation to failure and ultimate tensile strength were improved greatly after the forging process. The elongation to failure of the BDF ZK40-0.4Sr increased from 4.5 ± 0.3% to 19.6 ± 0.7%. The ultimate tensile strength of the BDF ZK40-1.6Sr increased from 88 ± 16.3 to 221.4 ± 5.7 MPa.

**Figure 7:**
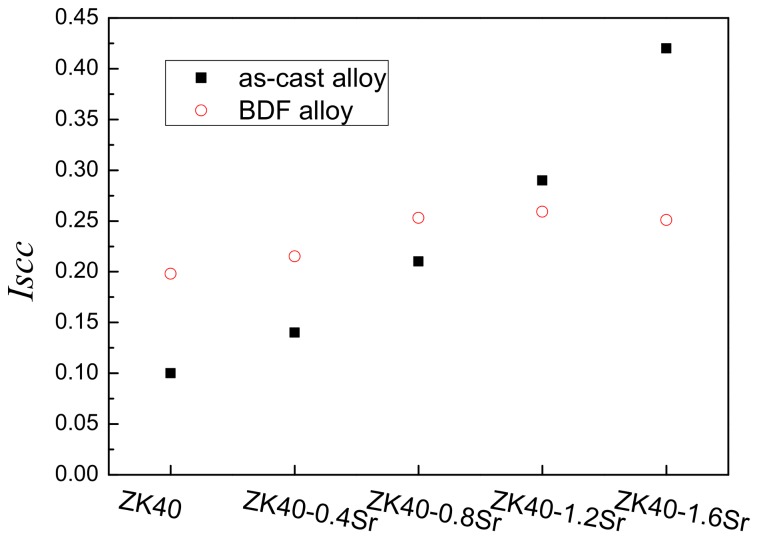
The comparison of the *I_SCC_* of the as-cast and BDF ZK40-xSr alloys.

Figure 7 presents a comparison of the SCC susceptibility indices (*I_SCC_*) measured for the BDF ZK40-xSr alloys with those of the as-cast ZK40-xSr alloys. With the increase of Sr addition, the *I_SCC_* of the as-cast ZK40-xSr alloys increased from *I_SCC_* = 0.10 to *I_SCC_* = 0.42. The SCC susceptibility indices of the BDF alloys increased from *I_SCC_* = 0.20 to *I_SCC_* = 0.25 (as listed in Table 2). The SCC susceptibility of the BDF alloys with less 0.8 wt.% Sr was higher than the corresponding value of the as-cast alloys, while the BDF alloys with more 0.8 wt % Sr was lower than that of the as-cast alloys. The results showed that the increase of SCC susceptibility due to the Sr addition was different in the as-cast and forged ZK40-xSr alloys.

### 3.4. Surface Appearance and Fractography

**Figure 8:**
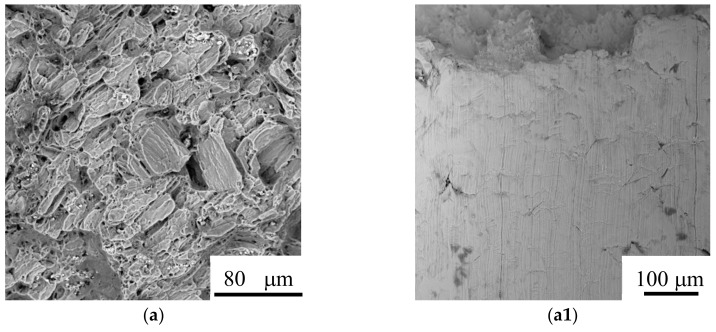

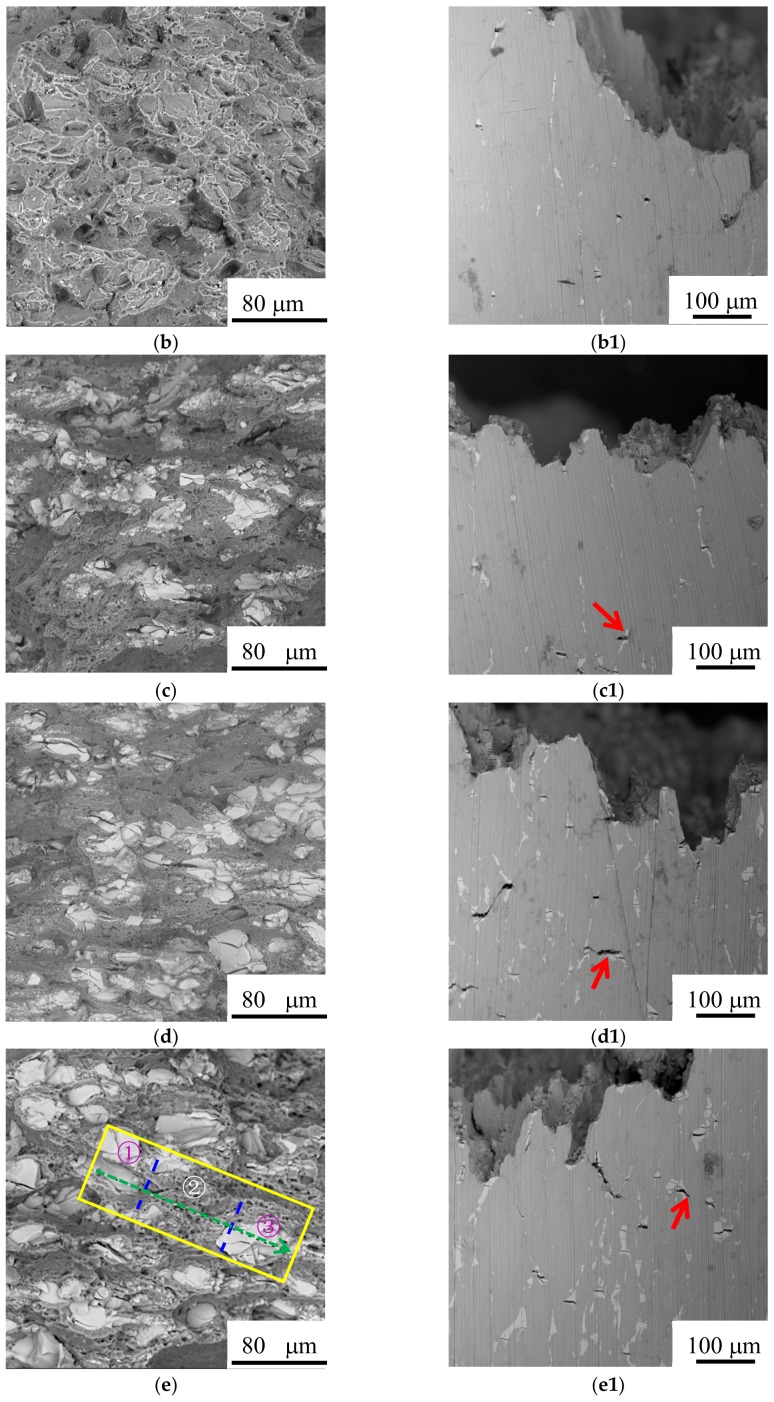

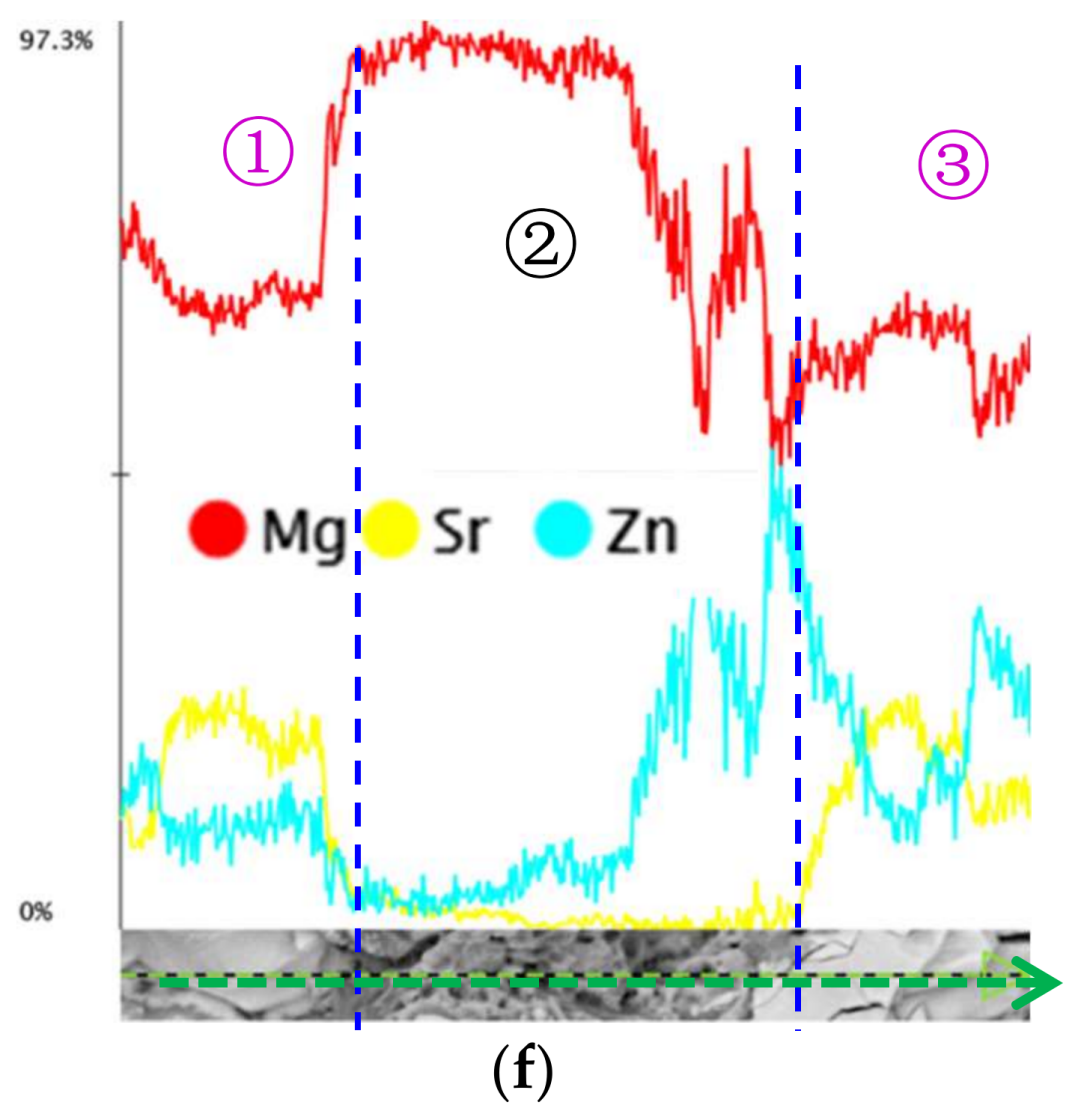
Fracture surfaces (backscattered electron images) of the BDF ZK40-xSr alloys tested in air: (**a**,**a1**) ZK40; (**b**,**b1**) ZK40-0.4Sr; (**c**,**c1**) ZK40-0.8Sr; (**d**,**d1**) ZK40-1.2Sr; (**e**,**e1**) ZK40-1.6Sr; (**f**) the energy-dispersive spectrometer (EDS) analysis of Figure 8e.

Figure 8 shows the fracture surfaces and fracture profile appearances of the BDF ZK40-xSr alloys tested in air at a strain rate of 10^−6^ s^−1^. Figure 8f shows the EDS analysis of the fracture features of the BDF ZK40-1.6Sr alloy. The bright areas are the second phase particles (including Mg, Sr and Zn) and the gray area is matrix Mg, as shown in Figure 8e,f. It could be seen that the BDF ZK40-xSr alloys showed a dimple feature in the matrix zone, and a cleavage fracture feature in the second phase area. Overall, the fracture surface of the BDF ZK40 and ZK40-0.4Sr alloys showed a ductile fracture feature. Dimples could be seen in Figure 8a,b. The fracture surface of the BDF ZK40-(0.8–1.6)Sr alloys revealed a mixed-mode fracture features, associating with both brittle fracture and dimple rupture, as shown in Figure 8c,d,e. With the increase of Sr addition, the brittle appearance features associated with the second phase particles increased, as shown in Figure 8c,d,e. The surface appearances of the BDF ZK40-xSr alloys tested in air were shown in Figure 8a1,b1,c1,d1,e1. Figure 8a1 indicated that there were some surface wrinkles on the surface of the BDF ZK40 alloy. However, there were some minor surfaces cracks of the second phase particles on the surface appearances of the BDF ZK40-xSr alloys containing Sr. With the increase of the second phase particles, the number and size of the cracks increased, as shown by arrows in Figure 8b1,c1,d1,e1.

**Figure 9:**
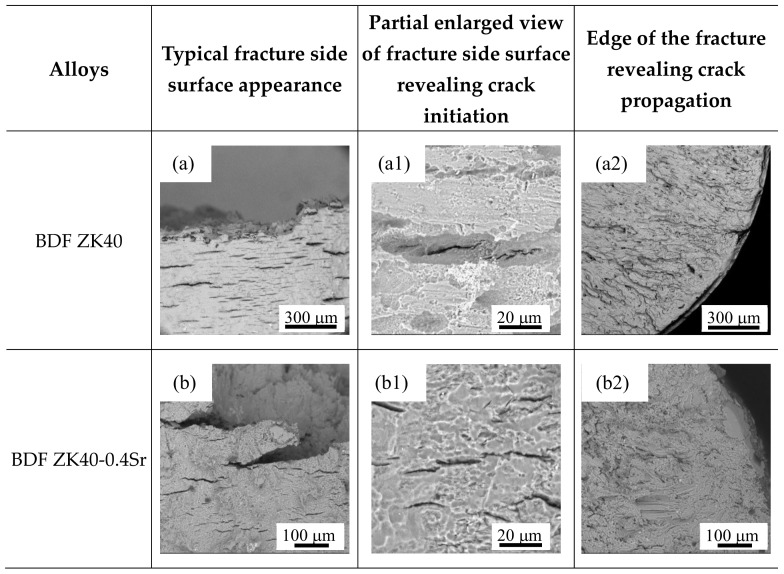

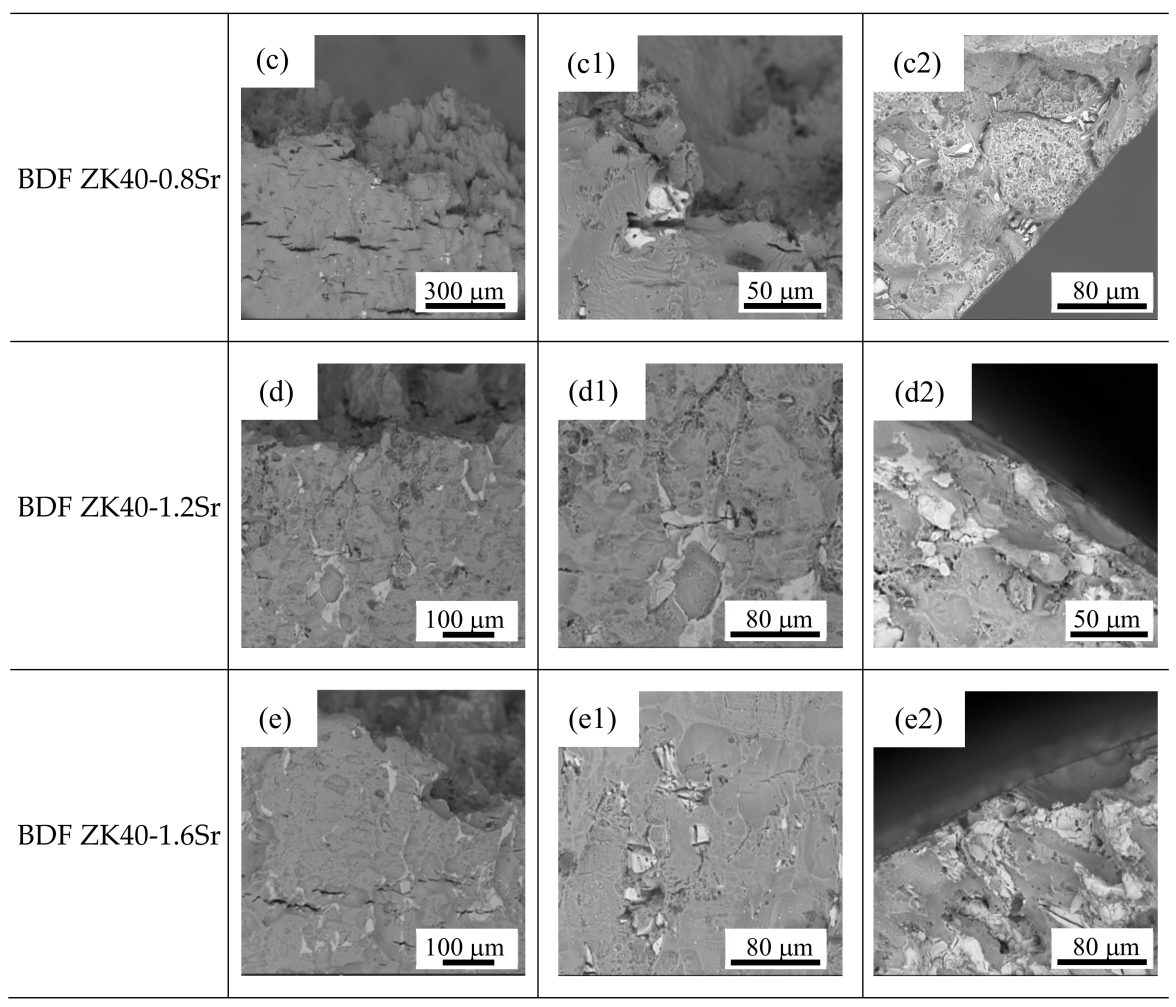
Scanning electron microscope (SEM) micrographs (backscattered electron images) of the BDF ZK40-xSr fractured surface in m-SBF: (**a**–**e**) typical fracture side surface appearance; (**a1**,**b1**,**c1**,**d1**,**e1**) Partial enlarged view of fracture side surface revealing crack initiation; (**a2**,**b2**,**c2**,**d2**,**e2**) edge of the fracture revealing crack propagation.

The fracture features of the BDF ZK40-xSr alloys tested in m-SBF are shown in Figure 9. The partial enlarged view of fracture side surface (Figure 9a,a1,b,b1) showed the minor cracks formed from the corrosion pitting for the lower Sr contained alloys (Sr = 0, 0.4 wt.%). However the number of cracks increased in the alloys with higher Sr contained, such as the BDF ZK40-1.2Sr/1.6Sr alloys. This may attributed to the brittle second phase particles (Figure 9d1,e1). The results indicated that there were micro-galvanic corrosion between the second phase and matrix, as shown in Figure 9e1. Both of the crack sources existed in the BDF ZK40-0.8Sr alloy (Figure 9c,c1). The fracture features of the BDF ZK40 and ZK40-0.4Sr alloy in Figure 9a2,b2 presented a broad and smooth plane, consisting of typical trans-granular cleavage features that resulted from the facture mode transformed from ductile to brittle fracture. The fracture surface of the BDF ZK40-(0.8-1.6)Sr alloy (Figure 9c2,d2,e2) revealed that the fractography was consistent with a mixed intergranular and trans-granular mode with trans-granular failure being predominant.

## 4. Discussion

### 4.1. Degradation Behavior and Mechanical Properties

It is known that the microstructure of Mg alloy can be manipulated by plastic deformation. In the present work, the forging process was adopted to refine the grain and improve the mechanical properties of the as-cast ZK40-xSr alloys. The average grain size of the as-cast alloys was about 100 µm, while that of the bi-directional forging (BDF) ZK40-xSr alloys was below 10 µm (as shown in Figure 2). The forging process was also effective in changing the distribution of second phase. For instance, the second phase was distributed along grain boundaries originally, and changed to islands or ellipsoidal shape uniformly dispersed between the grains in the BDF ZK40-xSr alloys (as shown Figure 1). The size of the second phase of the BDF ZK40-xSr alloys was larger than the average grain size of the matrix after the plastic deformation process. It was reported that the Mg_70_Zn_25_Sr_5_ or Mg_71_Zn_23_Sr_6_ was detected in the solution heat treatment Mg-4Zn-0.5Sr alloy [46]. The second phase still contained Mg, Zn and Sr element in the hot rolled Mg-4Zn-1Sr alloy [47]. XRD results indicated that the second phase could be a thermal-stable second phase. 

The corrosion resistance of biomedical magnesium alloys could be improved by plastic deformation [10,17]. Wang et al. [17] investigated the as-cast, rolled, and equal channel angular pressing (ECAP) AZ31 magnesium alloys (the average grain size was 450 µm, 20 µm, and 2.5 µm, respectively). They found that the degradation rate of the as-cast alloys was greater than that of the rolled and ECAPed alloys. In other Mg alloys, a similar trend was found [13]. The degradation rate of the extruded alloys was lower than that of the as-cast alloys. The improvement of the corrosion resistance of the Mg alloys was attributed to the grain refinement, the increase of the grain boundaries, and the homogenized redistribution of the second phase. In the present study, the corrosion resistance of the BDF ZK40-xSr alloys was also found improved after the plastic formation. The redistribution of the second phase and the reduction of grain size (from 8.2 ± 5.28 to 3.7 ± 1.91 µm) both contributed to the improvement of the corrosion resistance in the BDF ZK40-xSr alloys. However, the micro-galvanic corrosion between the second phases and the matrix increased, due to the number of the second phase increased with the increase of Sr content. That would deteriorate the corrosion resistance of the BDF ZK40-xSr alloys. In our experiments, the influence of micro-galvanic corrosion on the corrosion resistance of the BDF ZK40-xSr alloys was lower than the as-cast alloys. This might come from the homogenized distribution of the second phase, which was consistent with the previous studies of Brar [46] and Li [48]. 

The refined grain and redistribution of the second phase would also affect the mechanical properties of the BDF ZK40-xSr alloys. Based on the Hall-Patch theory, dislocations need to propagate from one grain to another through grain boundaries. Grain refinement leading to the increase of the grain boundaries would result in the difficulty of dislocation propagation, thus the strength of the grain refined alloys increase. Grain refinement could also improve the plasticity of the alloys [49]. The grain refinement of magnesium alloy can increase or activate the basal and angle cone slip, facilitate the grain boundaries sliding and rotation, shorten the moving distance of dislocation, and thus greatly improve the plastic deformation ability of the magnesium alloy. Therefore, the tensile strength and elongation to fracture of the refined BDF ZK40-xSr alloys improved significantly, as shown in Figure 6. However, the distribution and the volume fractions of the second phases, especially brittleness or toughness, would decrease the ductility of the magnesium alloys. It was reported that the large brittle second phases ruptured firstly under the tensile stress, thus led to the sharp decrease of the plasticity of the magnesium alloy. The fine second phase might interact with dislocation, thereby greatly improve the strength of the alloys. Therefore, the refined grain BDF ZK40-0.4Sr alloy with second phase pinning effect obtained the maximum tensile strength and elongation to failure in the BDF ZK40-xSr alloys. The tensile fracture morphology of samples tested in air showed that the second phase fracture was cleavage brittle fracture, while the matrix was ductile fracture (as shown in Figure 8). The surface appearances suggested the fracture source coming from the second phase. The results indicated that the plasticity of the alloys decreased with the increase of Sr content, due to the fact that the second phase was brittleness.

### 4.2. Stress Corrosion Cracking (SCC) Susceptibility

The SCC susceptibility of the magnesium alloys was affected by the grain size and the distribution of the second phase. Makar et al. [50] investigated the stress corrosion cracking (SCC) of the rapid solidification (RS) and as-cast Mg-1Al and Mg-9Al magnesium alloy in chloride solution. They found that RS and as-cast magnesium alloy have the same corrosion current density, while RS alloy have the better stress corrosion resistance due to the homogenization of composition and microstructure obtaining higher re-passivation rate. 

In the as-cast ZK40-xSr alloys, the fracture features revealed that primary intergranular SCC, which was attributed to the micro-galvanic corrosion between the net-like second phases and the matrix Mg. The micro-galvanic corrosion along the grain boundaries was the major factor affecting the SCC susceptibility of the as-cast alloys. Ben-Hamu et al. [51] and Kannan [52] also found that the intergranular SCC path were associated with the second phase particles along grain boundaries, while the trans-granular SCC fracture features in ZSM620/ZE41 was consistent with a mechanism involving hydrogen embrittlement. 

In the SSRT tests of the BDF ZK40 and ZK40-0.4Sr alloy, the trans-granular fracture characteristics, consistent with smooth fracture planes and the pitting crack sources, associated with the hydrogen embrittlement. The fracture mode transformed from ductile fracture to brittle fracture, and the SCC susceptibility of the BDF ZK40 and ZK40-0.4Sr alloy was higher than that of the as-cast alloys. It was reported that trans-granular fracture patterns consisted of planes, which were parallel to the propagation direction of cracks [22]. Argade et al. [40] found that the grain refined AZ31 alloy showed higher SCC susceptibility. It was attributed to the enhanced hydrogen diffusivity. The kinetics of hydrogen diffusion through grain boundaries was expected to be higher, as the finer grain microstructure had higher grain boundary area per unit volume [21]. Song et al. [53] found the pre-exposure embrittlement (i.e., the ductility loss) increased with the increase of the pre-exposure time. Hydrogen can decrease the cohesive strength of Mg, hence cause hydrogen embrittlement [54]. 

In the present work, it was found that the grain refinement and homogenized second phase distribution in the BDF alloys could suppress the adverse effect of Sr on the SCC susceptibility. Compared with the SCC susceptibility of the as-cast ZK40-xSr (x > 0.8 wt %) alloys, that of the BDF ZK40-xSr alloys shows a slight fluctuation, even decrease. The BDF ZK40-0.8Sr alloy showed no larger hydrogen embrittlement fracture plane, the same intergranular corrosion cracking features and slightly higher stress corrosion susceptibility than that of the as-cast alloys. These uniformly distributed island or elliptic shaped phases in the BDF ZK40-1.2Sr and ZK40-1.6Sr alloy had high volume fraction, which could avoid the micro-galvanic corrosion along the grain boundaries. Thus, it would suppress the hydrogen embrittlement and the beginning of the intergranular SCC. Cao [41] investigated the SCC properties of solid solution treated and hot rolled Mg-X (X = Zr, Sr, Ce, Nd, Gd, and Ca) alloys. They found that rolling processing caused the grain refinement, reduced the size of the second phases, and eliminated defects such as porosity or shrinkage holes, thus improving the stress corrosion resistance performance of the alloys. Hakimi [55] investigated the influence of grain refinement on the corrosion resistance of Mg-6%Nd-2%Y-0.5%Zr (EW62) alloy. Their results showed that the corrosion resistance and stress corrosion resistance of EW62 after extrusion improved compared with the as-cast EW62 alloy.

## 5. Conclusions

In order to study the effect of microstructure and second phase on the stress corrosion cracking (SCC) susceptibility of the forged ZK40-xSr magnesium alloys, immersion testing, electrochemical analysis, and slow strain rate tensile (SSRT) tests were carried out. The main conclusions are shown as follows:

(1) The average grain size changed from about 100 µm (as-cast ZK40-xSr alloys) to below 10 µm after the forging process. The average grain size of the BDF ZK40-0, 0.4, 08, 1.2, and 1.6Sr alloys were 8.2 ± 5.3, 6.7 ± 5.3, 5.7 ± 4.3, 3.7 ± 2.0, and 3.7 ± 1.9 µm, respectively. The forging process also changed the distribution of the second phase from net-shape along the grain boundaries to uniformly island shape inside the Mg matrix.
(2) The mechanical properties of the BDF ZK40-xSr alloys improved significantly, and the corrosion resistance enhanced after the forging process. These were attributed to the grain refinement and uniformly distribution of the second phase.
(3) The micro-galvanic corrosion along the grain boundaries was the major factor affecting the SCC susceptibility in high Sr ZK40-xSr alloys. The hydrogen embrittlement increased the SCC susceptibility of the low Sr alloys (BDF ZK40 and ZK40-0.4Sr). However, the forging process can suppress the adverse effect of Sr on the SCC susceptibility of the magnesium alloy, owing to the uniformly distributed second phase and refined grains.

## Figures and Tables

**Table 1:** Corrosion potential, corrosion current density, and degradation rate for the as-cast and BDF ZK40-xSr alloys in m-SBF at 37 °C.

Materials		Corrosion Potential, E_corr_ (V)	Corrosion Current Density, i_corr_ (mA·cm^−2^)	Degradation Rate (mm·y^−1^) by polarization CURVES	Degradation Rate (mm·y^−1^) by H_2_ Evolution
As-cast	ZK40	−1.770	0.255	5.83 ± 0.27	4.59 ± 0.30
	ZK40-0.4Sr	−1.767	0.266	6.08 ± 0.22	4.78 ± 0.21
	ZK40-0.8Sr	−1.788	0.343	7.84 ± 0.31	6.47 ± 0.38
	ZK40-1.2Sr	−1.791	0.445	10.17 ± 0.34	8.31 ± 0.31
	ZK40-1.6Sr	−1.805	0.558	12.75 ± 0.41	10.34 ± 0.29
BDF	ZK40	−1.706	0.207	4.73 ± 0.20	3.82 ± 0.13
	ZK40-0.4Sr	−1.665	0.195	4.46 ± 0.18	3.51 ± 0.19
	ZK40-0.8Sr	−1.620	0.216	4.94 ± 0.14	3.99 ± 0.17
	ZK40-1.2Sr	−1.605	0.236	5.39 ± 0.21	4.34 ± 0.13
	ZK40-1.6Sr	−1.615	0.242	5.53 ± 0.16	4.61 ± 0.15

**Table 2:** Summary of SSRT results and the *I_SCC_* of the BDF ZK40-xSr alloys.

Alloys	*ε*_in air_/%	*ε*_in m-SBF_/%	UTS _in air_/MPa	UTS _in m-SBF_/MPa	*I* *_SCC_*
BDF ZK40	15.3 ± 0.6	7.8 ± 0.3	226.3 ± 4.9	194.5 ± 6.1	≈0.20
BDF ZK40-0.4Sr	19.6 ± 0.7	9.5 ± 0.5	261.1 ± 6.3	224.2 ± 7.7	≈0.22
BDF ZK40-0.8Sr	14.5 ± 0.5	5.3 ± 0.2	248.5 ± 2.8	202.1 ± 9.4	≈0.25
BDF ZK40-1.2Sr	9.8 ± 0.4	4.2 ± 0.4	234.2 ± 7.8	183.3 ± 2.1	≈0.26
BDF ZK40-1.6Sr	6.6 ± 0.3	3.2 ± 0.3	221.4 ± 5.7	171.3 ± 4.3	≈0.25
